# Implantable loop recorder migration as cause of unexplained chronic chest pain

**DOI:** 10.1093/ehjcr/ytaf259

**Published:** 2025-05-23

**Authors:** Niels Verlinde, Jan Van Keer, Roel Beelen

**Affiliations:** Department of Thoracovascular Surgery, OLV Aalst, Moorselbaan, Aalst 164 9300, Belgium; Department of Cardiology, Heartcenter OLV Aalst, OLV Aalst, Moorselbaan, Aalst 164 9300, Belgium; Department of Thoracovascular Surgery, OLV Aalst, Moorselbaan, Aalst 164 9300, Belgium

## Case Report

**Figure 1:**
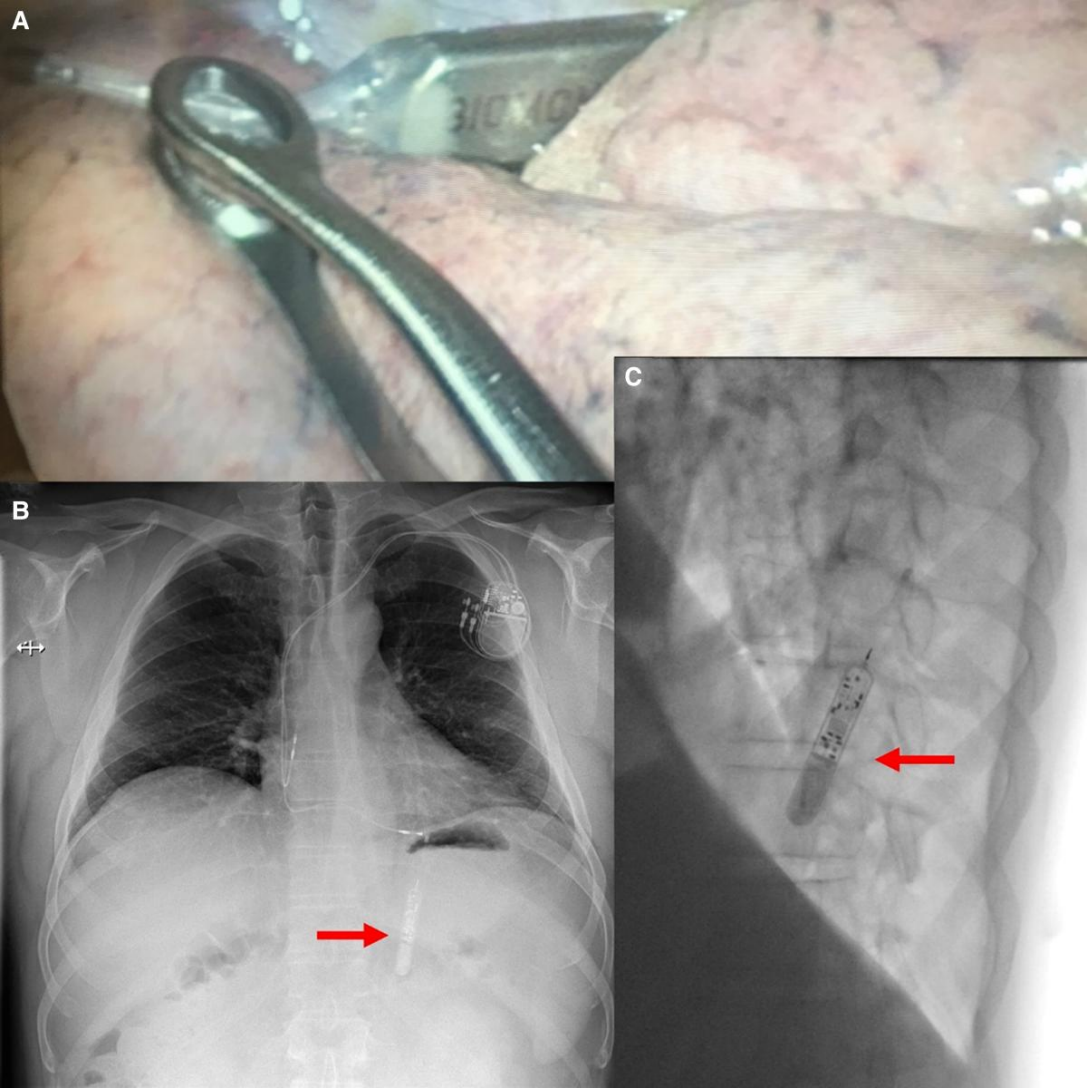
(*A*) Thoracoscopy with intrapleural loop monitor; (*B*) chest X-ray face (first post-implantation imaging); (*C*) chest X-ray profile (Pre-operative imaging).

A 55-year-old man developed chronic pleuritic chest pain in the months after the implantation of a Biotronik Biomonitor III loop recorder. Chest X-ray showed a migrated loop recorder located in the subpleural space (*Figure 1C*).

The device was removed by thoracoscopy 1 year after loop recorder implantation, with a resolution of the chest pain (see *Figure 1A*). Of note, shortly after the loop recorder implantation, the patient received chest massage and pacemaker implantation because of sinus arrest with haemodynamic collapse. Chest X-ray post pacemaker implantation still showed the correct position of the loop recorder (*Figure 1B*). Interestingly, the patient had received another loop recorder, which was explanted 10 years prior.

Migration of a loop recorder is a rare complication, mostly occurring shortly after implantation.^1^ The patient’s body habitus (muscular chest wall), history of prior loop recorder implantation at the same site, and chest compressions during cardiopulmonary resuscitation, might have played a role.

A proper implantation technique with minimal angle of penetration (to ensure subcutaneous instead of muscular placement) is advised to prevent this complication.^2^ Clinical follow-up with device interrogation and telemonitoring are recommended to recognize migration in an early stage.^1,3^

**Consent:** Patient declared consent for scientific research, according to COPE guidelines.

## Data Availability

The data underlying this article will be shared on reasonable request to the corresponding author.
